# ClearedLeavesDB: an online database of cleared plant leaf images

**DOI:** 10.1186/1746-4811-10-8

**Published:** 2014-03-28

**Authors:** Abhiram Das, Alexander Bucksch, Charles A Price, Joshua S Weitz

**Affiliations:** 1 School of Biology, Georgia Institute of Technology, Atlanta, Georgia, USA; 2 School of Physics, Georgia Institute of Technology, Atlanta, Georgia, USA; 3 School of Interactive Computing, Georgia Institute of Technology, Atlanta, Georgia, USA; 4 School of Plant Biology, University of Western Australia, Perth, Australia

**Keywords:** Database, Images, Leaves, Digital archiving, Digital curation

## Abstract

**Background:**

Leaf vein networks are critical to both the structure and function of leaves. A growing body of recent work has linked leaf vein network structure to the physiology, ecology and evolution of land plants. In the process, multiple institutions and individual researchers have assembled collections of cleared leaf specimens in which vascular bundles (veins) are rendered visible. In an effort to facilitate analysis and digitally preserve these specimens, high-resolution images are usually created, either of entire leaves or of magnified leaf subsections. In a few cases, collections of digital images of cleared leaves are available for use online. However, these collections do not share a common platform nor is there a means to digitally archive cleared leaf images held by individual researchers (in addition to those held by institutions). Hence, there is a growing need for a digital archive that enables online viewing, sharing and disseminating of cleared leaf image collections held by both institutions and individual researchers.

**Description:**

The Cleared Leaf Image Database (ClearedLeavesDB), is an online web-based resource for a community of researchers to contribute, access and share cleared leaf images. ClearedLeavesDB leverages resources of large-scale, curated collections while enabling the aggregation of small-scale collections within the same online platform. ClearedLeavesDB is built on Drupal, an open source content management platform. It allows plant biologists to store leaf images online with corresponding meta-data, share image collections with a user community and discuss images and collections via a common forum. We provide tools to upload processed images and results to the database via a web services client application that can be downloaded from the database.

**Conclusions:**

We developed ClearedLeavesDB, a database focusing on cleared leaf images that combines interactions between users and data via an intuitive web interface. The web interface allows storage of large collections and integrates with leaf image analysis applications via an open application programming interface (API). The open API allows uploading of processed images and other trait data to the database, further enabling distribution and documentation of analyzed data within the community. The initial database is seeded with nearly 19,000 cleared leaf images representing over 40 GB of image data. Extensible storage and growth of the database is ensured by using the data storage resources of the iPlant Discovery Environment. ClearedLeavesDB can be accessed at http://clearedleavesdb.org.

## Background

The leaf organ is the site of the majority of primary production within land plants. Leaves vary in shape, composition, color, as well as the structure of their venation networks [1]. Here, we focus on efforts to describe and analyze the last of these characteristics: the venation network. A leaf venation network represents the interconnected set of veins present in a leaf. Carbon, nutrients, and water are transported through xylem and phloem contained within the vascular bundles that constitute veins [2]. In addition, veins confer material strength to the leaf due to the higher stiffness of veins relative to other leaf tissue [3,4].

The analysis of leaf venation networks is of increasing interest due to multiple hypotheses linking venation networks to changes in leaf physiological rates [5,6] and to the diversification of early land plants [7-9]. For example, increases in the density of leaf veins are hypothesized to increase physiological rates, such as net photosynthesis, because of the decreasing distance that water molecules must travel between the vein network, terminal sites of photosynthesis and stomates [10-12]. Similarly, the early adaptive radiation of land plants coincided with the appearance of increasingly reticulate (i.e. loopy) venation networks, as inferred from fossil leaves [13,14]. Reticulate networks are known to confer added redundancy to leaves in the face of damage from herbivory or disease, providing a mechanism linking leaf venation structure to whole plant fitness [15].

In practice, the quantitative and qualitative analysis of venation networks requires the identification of veins as distinct from that of surrounding leaf tissue in areoles. Hence, a first step toward identifying a venation network is to “clear” a leaf [16,17]. Multi-decadal efforts to collect cleared leaves have been undertaken by individual researchers and institutions. For example, in the USA there are now multiple, independent cleared leaf collections with thousands of specimens, e.g., held at the Smithsonian Institution [18], Yale Peabody Museum [19], New York Botanical Garden [20], and UC-Berkeley [21]. In each case, digital images have been taken of the specimens and in many instances, institutional websites offer free access to all or a portion of these curated collections. However, these sites do not necessarily provide functionality for individual researchers unaffiliated with these institutions to store, manage, search, and analyze images online – indeed, such functionality is outside of the scope of each individual collection. There are also other generalized repositories of images of plants and plant organs such as CalPhoto [22], Morphbank [23], USDA Plant database [24], Encyclopedia of Life records [25] and Bisque [26]. Bisque is notable for its support for secure image storage, analysis and data management capabilities. Bisque is also hosted on the iPlant [27] environment giving users control over sharing of images and analysis results. While these repositories are freely accessible, they are quite general in scope and are not designed for the specific set of features and meta-data associated with cleared leaf images. Moreover, individual collection sites are not linked, they do not share a common platform to share data, and they do not enable the integration of cleared leaf images with processed images and analysis. Hence there is a need for a complementary approach to enhance the utility of pre-existing digital-physical collections: a trans-institutional online database by which individual research groups and institutions can store, view, and share their cleared leaf images with the community.

In order to address these issues, we have developed the Cleared Leaf Image Database (ClearedLeavesDB). ClearedLeavesDB enables researchers to contribute, access, and share cleared leaf images. The database houses existing curated collections, such as a major contribution from the Smithsonian Institution collection, and enables researchers to contribute small-scale curated collections for dissemination, viewing and re-use. The web interface of ClearedLeavesDB is built on open-source technologies, allowing users to: (i) create and manage leaf image collections; (ii) upload/download images and their metadata in single or batch mode; (iii) annotate images; (iv) search images; and (v) link analysis data with the original images. The database provides varying degree of access based on user privileges which enhances data quality and reliability. As we describe below, we have developed ClearedLeavesDB to accelerate basic science and public understanding of plant biology generally, and leaf veins specifically.

### Construction and content

We describe a user-oriented view of the ClearedLeavesDB system, given that our focus in this paper is on the practical usage of the web application by and for plant biologists. Major sections of the application are described below, including how to create and manage image collections, search and browse images, manage and download marked lists of images, and user roles. For users interested in viewing and modifying the source code, we note that the application is developed using Drupal [28]; a content management system built upon Apache web server [29], MySQL [30] database server and PHP [31].

#### Create and manage collections

**Figure 1:**
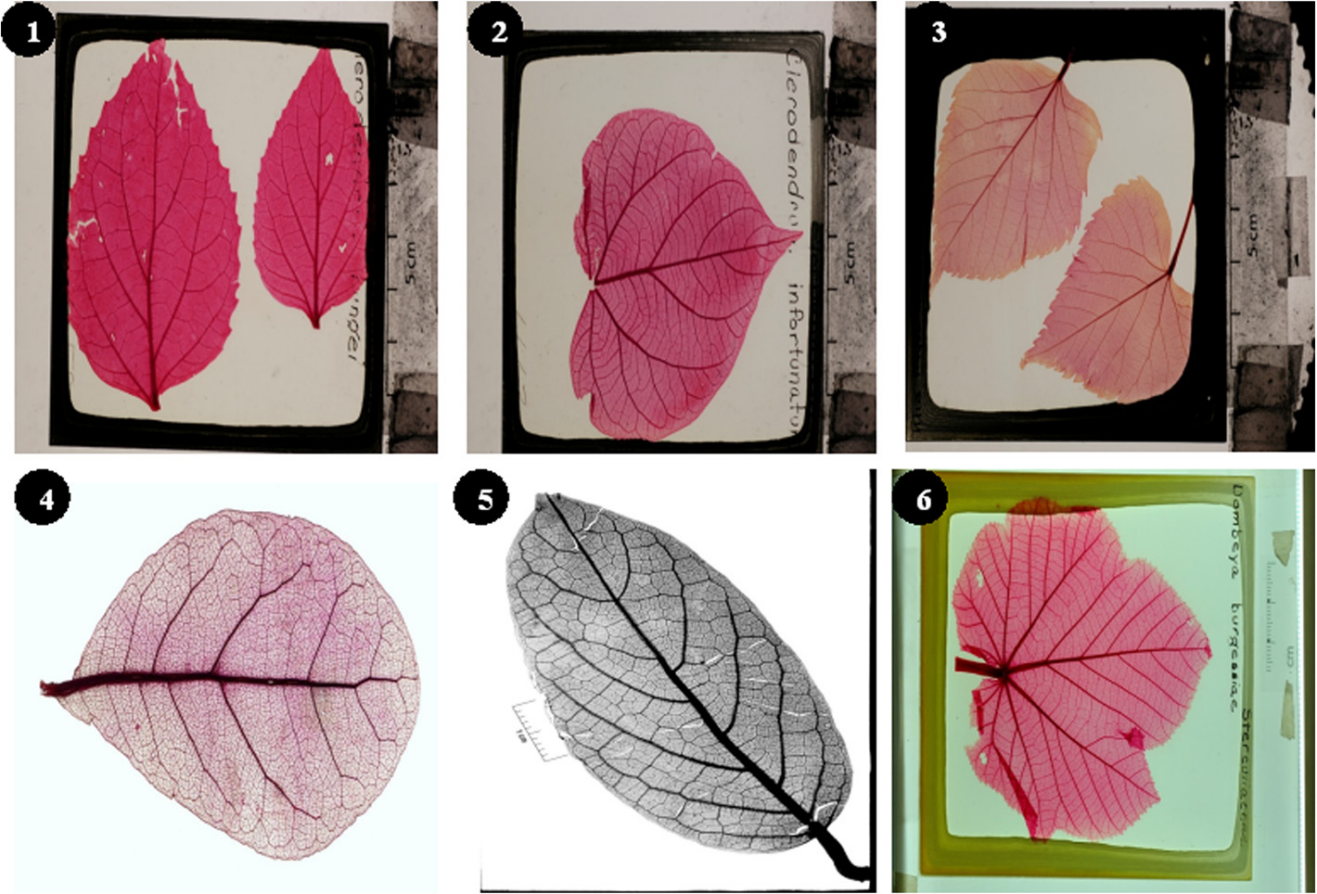
**Representative images of cleared leaves in ClearedLeavesDB. 1**. *Clerodendrum bungei Steud*. **2**. *Clerodendrum infortunatum L*. **3**. *Tilia miqueliana Maxim*. **4**. *Quercus laurifolia ***5**. *Pseudocalyx africanus S. Moore ***6**. *Dombeya burgessiae*

**Figure 2:**
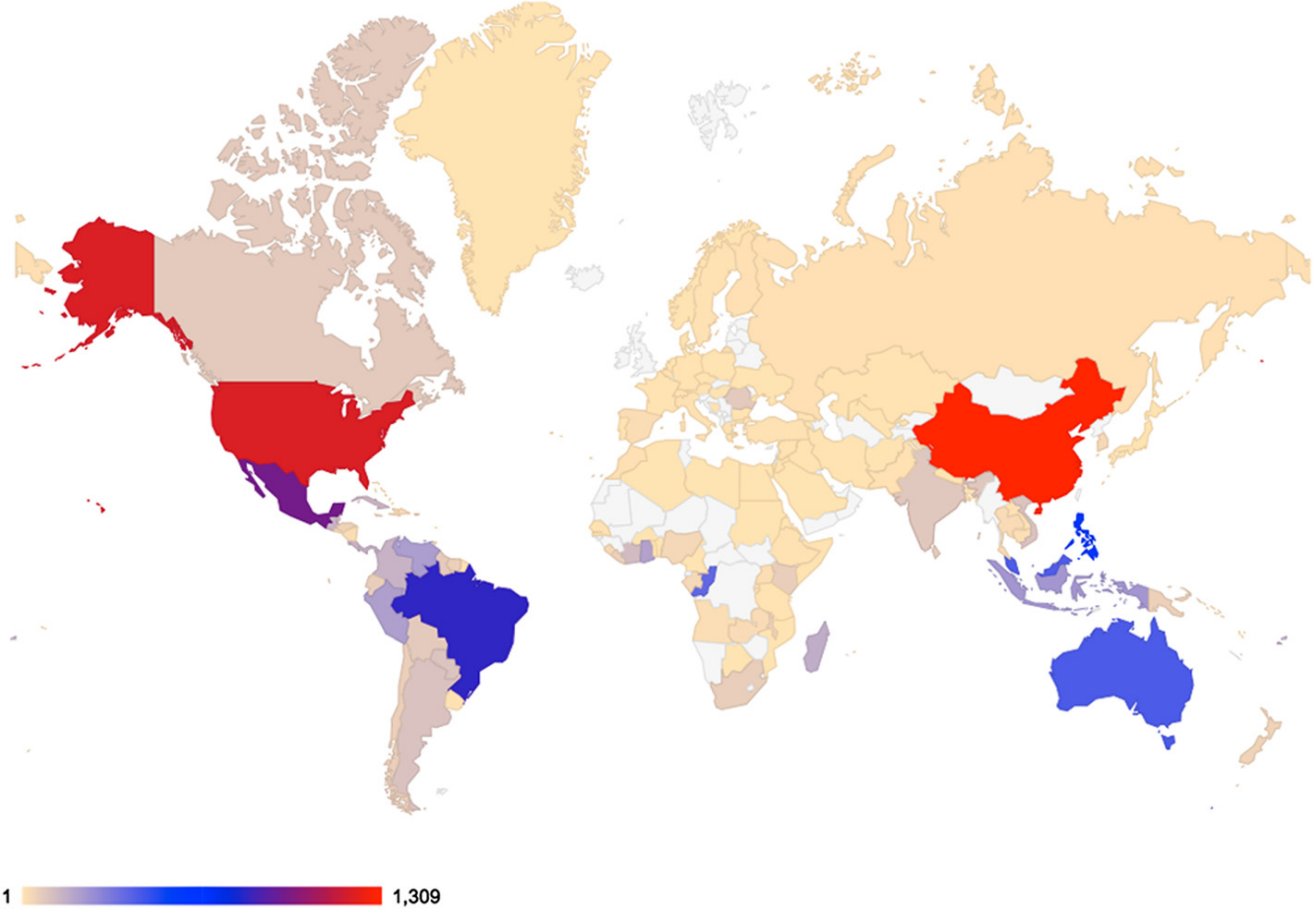
**Geographic distribution of source specimens in the public collections of ClearedLeavesDB.** Each country’s color denotes the number of specimens for which images are available in the database as of submission. The world map projection is via Google geo chart: https://code.google.com/apis/ajax/playground/?type=visualization#geo_chart.

Image collections are sets of uploaded images that share some common attributes (e.g. geographical location, experimental treatment, species etc.). These collections are curated and linked to the owner of the collection (but stored centrally). While creating a collection, the owner has to provide a name and brief description of the collection. The owner also has the option to mark a collection as private or public and the license under which the data is released. Public collections can be accessed by all users and visitors of the database whereas private collections can only be accessed by the owner and associated members. A personalized access module allows the owner to manage a collection membership for all registered users (see the user roles section below for details). Once a collection is created, images are uploaded via a simple user interface. Users with required privileges are permitted to annotate images. The collection owner can change all initial preferences at any time via the “Collections” menu. At the time of submission, ClearedLeavesDB includes nearly 19,000 images (see Figure 1 for a sample of the images available) from over 144 countries (see Figure 2 for the current geographical distributions of source specimens). Additional, global information on the collections can be viewed via the “Charts” menu.

#### Search and browse images

**Figure 3:**
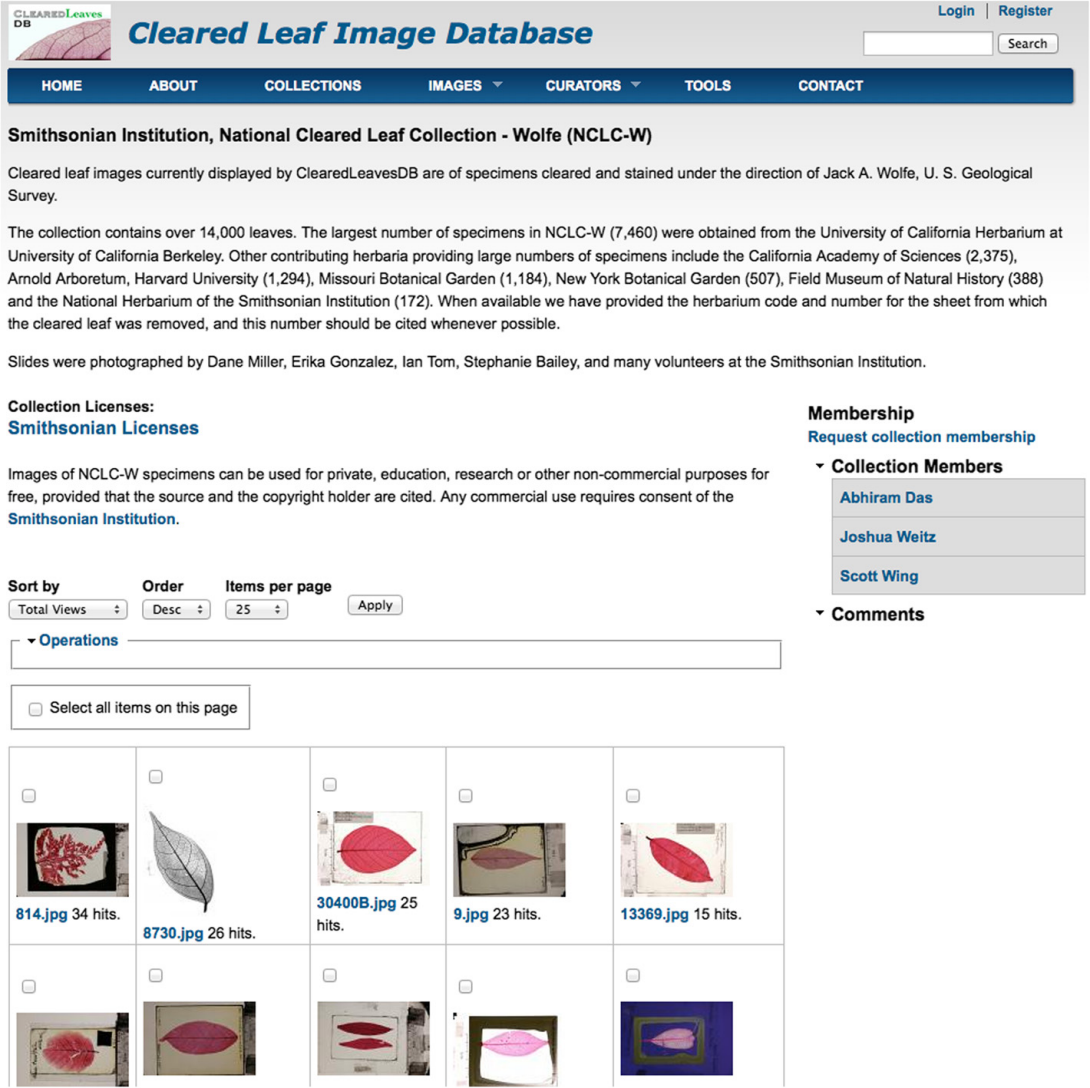
**Collection view for curator user.** Curator has the right to add image to the marked list, post comments on the collection or manage collection membership.

A user searches for images or collections via a name or various metadata parameters. Current search fields include: class, order, family, genus, species, blade margin, blade shape, herbarium ID, identified by, specimen number, image resolution and whether or not the original image has processed images associated with it. A free text search is also available. When viewing a collection (Figure 3) a user sees thumbnails of all images. A mouse click on the thumbnail leads to a detailed image view with metadata. The search functionality is accessible via a form available in the “Images” menu.

#### Marked list

**Figure 4:**
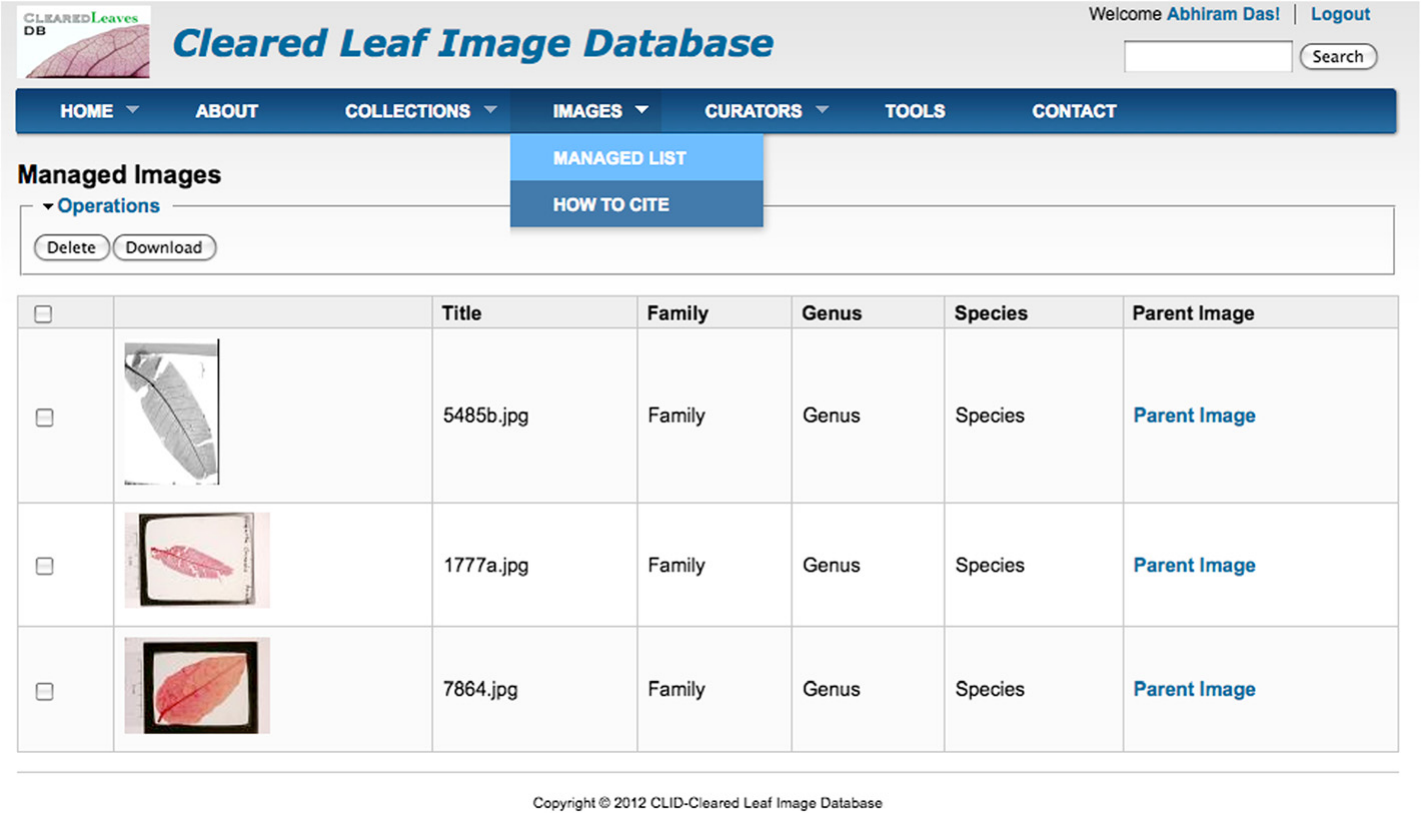
**Marked list of images.** User can bulk download images from the marked list with its metadata.

A marked list allows the user to maintain a list of images of interest as they search or browse. A user can add images to the marked list from the collection view module or via the search module. The marked list module also allows the user to download image groups (Figure 4) together with their associated metadata as a “zip” file. The downloaded zip file contains a comma-separated value (.csv) file containing the image name associated with the image metadata – such formats are compatible with all standard spreadsheet programs. Only registered users of the system have the privilege to maintain a marked list and batch download images. The metadata file contains a unique number i.e. the database identifier for each image downloaded. This number is required to associate processed images and their analysis data to the parent image in the database while uploading processed images through our desktop client interface and API. Users interested in batch uploading processed images to the database are advised to use the batch download functionality to obtain image metadata. To facilitate this, we introduce a java tool for automated external access to the database without using the web interface (described next).

#### Interface for linking processed data with original images

ClearedLeavesDB is capable of storing original and processed images along with their metadata. The types of processed images might be binary images resulting from manual or automated segmentation of leaf veins from the leaf background as in [5,6], skeletonized images of the approximate center-line of the leaf venation network [5], or aereole segmentation of the image [5]. Additional processed data might include the results of quantitative analysis of the structure of the cleared leaf. In order to link post-processed images and data to the original image, we provide tools to upload processed images and its analysis data to the database from a user’s computer through a command line interface and application programming interface (API).

The command line interface is a Java client application that can be downloaded from the ClearedLeavesDB website (http://www.clearedleavesdb.org/sites/default/files/clid.jar). The downloaded file “clid.jar” is an executable Java Archive (jar) file that contains the client application and an open API. This application and API uses Representational State Transfer (REST) Web services to connect and upload processed data to the database. It requires Java Runtime Environment (JRE) version 1.6 or above to be installed on the user’s desktop or laptop. Instructions on how to launch the command line application is described in detail on the web site and can be accessed by going to the “API” tab of the “TOOLS” menu. Database users can also upload processed images and its analysis data through the web interface. To do so, users should navigate to the image view page from search, collection view or marked list page and click on “Add new processed image” link. The API is provided through a Java class called “UploadProcessedImages”. It exposes an interface called “ToCLID()” that requires three parameters:

1. CSV file name (String data type)

2. Database user name (String data type)

3. Database user password (String data type)

The following Java code snippet can be used to invoke the API:

import clid.rest.client.UploadProcessedImages;

UploadProcessedImages.ToCLID(csvFile, username, password);

Please note that the CSV file should be compatible to the format as mentioned on the website. A sample file can be downloaded from ClearedLeavesDB website (http://www.clearedleavesdb.org/sites/default/files/sample_processed_upload.csv).

Analyzing images with third party software such as LeafGUI [32], LAMINA [33] and LIMANI [34] is enabled by the integrated batch download functionality. After analysis, processed images and their corresponding analysis data can be uploaded. These uploaded data are associated with the original image, hence heterogeneous and non-standard processing types can be uploaded, though the processed analysis data cannot yet be searched, a trade-off given the design decision to allow greater flexibility in allowing users to associate processed data with the original images.

#### User roles

The database provides multiple levels of user access to cater to different needs of the community. The database provides the following three levels of users (see Table 1 for a list of user roles and associated functions): 1) guests, 2) registered users and 3) curators. A collection manager (i.e. a curator) can define custom roles and manage permissions for the collection irrespective of the global roles defined above. They can do so by clicking on the “Group” tab of the collection.

**Table 1:** List of user roles and their associated permissions

**Permissions**	**Guest**	**Registered User**	**Curator**
Browse collections and images	√	√	√
View collections and its metadata	√	√	√
View images and its metadata	√	√	√
View high resolution original images	Χ	√	√
Create image collections	Χ	√	√
Upload images to the collection	Χ	√	√
Bulk upload images to the collection	Χ	Χ	√
Upload metadata to the collection	Χ	√	√
Comment on collections and images	Χ	√	√
Maintain a marked list of images	Χ	√	√
Download images and its metadata from the marked list	Χ	√	√
Image upload quota	0 KB	1 GB	20 GB
Add processed images and results	Χ	√	√

### User scenarios

In this section we describe several examples to highlight the main use cases of our database: database browsing by a guest user; creating an image collection; managing collection membership, roles and permissions; and uploading processed images through the REST Web Services client.

#### A guest browsing the database

A user accessing the database will first arrive on the home page displaying the welcome message and a list of public collections available on the database. The user can either select any one of the collections shown or click on the “Collections” menu to search for a public collection and then click on it to browse the collection. The collection view (Figure 3) displays thumbnail images in the collection in a grid format along with members of the collection and comments posted by different users. The guest user does not have rights to post comments, add images or request membership to the group. Guests can select an image and view its metadata but cannot view full size images, post comments on an image or edit metadata. Guests can click on the “Images” menu to search for images with certain filters. From the image search page user can also navigate to the image details view page as described earlier. Guests do not have access to the marked list functionality i.e. adding images to the marked list from collection or search page.

#### Registered user and curator creating image collection

Here we describe the difference in creating an image collection by an authenticated user and a curator. Both these user roles have the rights to create image collections but registered users do not have rights to bulk upload images to the collection. We demonstrate this functionality using a registered user and a curator. Both these users have access to the “Create Collection” sub-menu in “Collections” menu on the database but the registered user does not have access to “Bulk Upload Images” sub-menu. After creating a collection, the registered user is redirected to the single image upload page whereas the curator is redirected to the bulk upload page.

#### Managing collection membership, roles and permissions

The founding curator (i.e., “author”) of a collection has all the rights to manage its members, roles and permissions. The author can visit the “Group” tab and click on the “Add People” link to add people to the collection and click on the “People” link to manage membership. Similarly “Roles” and “Permissions” link can be followed to manage roles and permissions of the collection.

#### Uploading processed images through REST web services client

**Figure 5:**
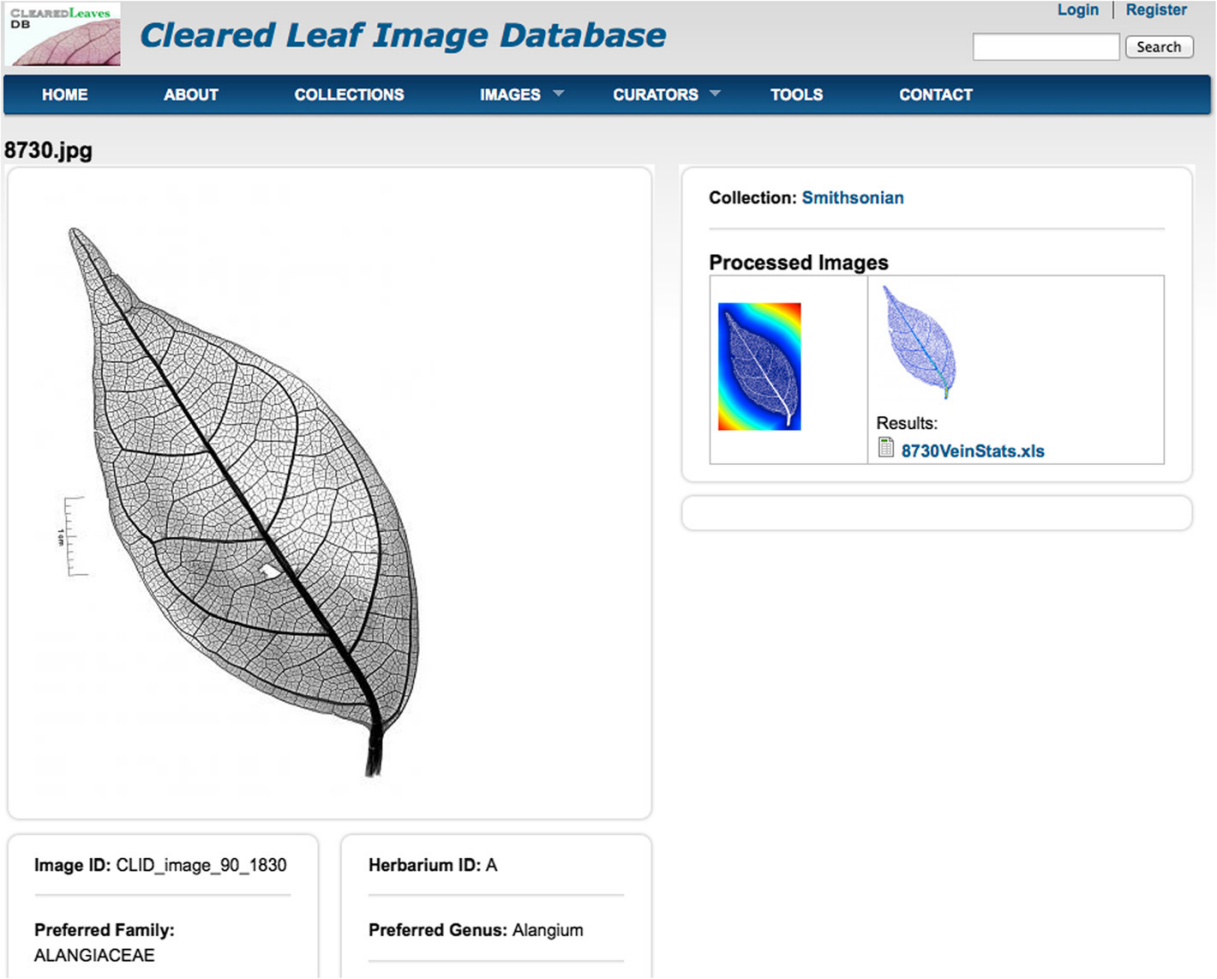
View of the image showing associated processed image and its analysis data.

Our client application can be used to upload processed images and its analysis data to the database. A registered user or a curator can add images to its marked list (Figure 4) and batch download the images with its metadata using the download feature. After the images are downloaded a user can use any image processing software to analyze image traits and produce processed images and analysis data files. These processed images and analysis data files can be uploaded to the database using the Web services client. The client application reads a csv file containing the parent image id and a corresponding processed image and data file. A sample csv file is available for download at the website in the FAQ section (i.e. CSV file format) of the ‘About’ page. The image name and ID in the csv file are obtained from the metadata file downloaded along with the images. In this case study, we downloaded three images from a user’s marked list (Figure 4), processed them using Leaf GUI [32] and uploaded the analysis data using the client application. A user can view the processed images and their analysis data by navigating to the parent image’s view page (Figure 5).

#### Datasets

ClearedLeavesDB is seeded with five distinct collections. The largest is from the National Cleared Leaf Collection housed at the Museum of Natural History, Smithsonian Institution, including specimens cleared and stained under the direction of Jack A. Wolfe, U.S. Geological Survey. The other collections are of (i) *Arabidopsis thaliana* specimens from a study of variation in venation network traits for which data include ecotypes, RILs, NILs, and vascular patterning mutants (courtesy of Benjamin Blonder, unpublished); (ii) specimens from multiple *Populus tremuloides* clones from the Colorado Rocky Mountains [35]; (iii) specimens taken from the University of Arizona arboretum [36]; (iv) specimens taken from oak trees of different species on the campus of the Georgia Institute of Technology (courtesy of Charles Price, unpublished).

## Discussions and conclusions

ClearedLeavesDB provides a range of practical tools to store, manage and access cleared leaf images. The web interface for the database is built on open source technologies and is freely accessible online. At present, the database is seeded with over 40 GB of primary images of cleared leaves representing a total of 19,000 images. In doing so, ClearedLeavesDB provides a means to connect researchers, institutional repositories, and the public in accessing, sharing, and analyzing the biology of plant leaves. In this sense, ClearedLeavesDB is complementary to pre-existing websites that enable access to images of plants and plant organs, and specialized repositories of cleared leaf images. ClearedLeavesDB aims to bridge the gap between these two types of platforms by offering individuals and institutions a common platform, built on open source technology, to store, manage, share, view and analyze cleared leaf images. At present, ClearedLeavesDB leverages the infrastructure of iPlant [27] for flexible storage and access of third-party developed tools. In moving forward, we plan to extend the current system to enable further, integrated analysis of cleared leaf images and associated trait data. First, we plan to enable the association of images stored on ClearedLeavesDB with trait data pertaining to the original leaf and plant specimens, e.g., as stored on TRY-db.org [37], a global database for plant traits. Second, we plan to extend the current system to integrate the database with iPlant’s Data Store [27] built using an integrated rule-oriented data-management system (iRODS) [38]. Hence, future efforts to analyze large-scale datasets of cleared leaves may benefit from bringing the software analysis tools to the data, rather than the other way around, as well as leveraging many of the other benefits of a scalable infrastructure.

### Availability and requirements

The database is named as “ClearedLeavesDB” and is accessible at http://clearedleavesdb.org. The database can be accessed through any web browser; however it has been tested on Firefox (Version 27.0.1), Chrome (Version 33.0.1750.117), Safari (Version 6.1.1 and Version 7.0.1) and Internet Explorer (Version 11.0.9.x). A quick start guide is available on the website and is included here as Supplementary File 1. The public datasets present in the database are freely accessible but carry copyright information defined by the collection author.

### Availability of supporting data

The data sets supporting the results of this article are available in the ClearedLeavesDB repository, http://clearedleavesdb.org.

## Abbreviations

REST: Representational State Transfer; JVM: Java Virtual Machine.

## Competing interests

The authors declare that they have no competing interests.

## Authors’ contributions

AD designed the database, developed the database and web interface, and co-wrote the manuscript. AB designed the database. CAP conceived of the project plan and contributed to writing the manuscript. JSW conceived of the project plan, contributed to web interface design, and co-wrote the manuscript. All authors read and approved the final manuscript.
